# Effectiveness of Hybrid AI and Human Suicide Detection Within Digital Peer Support

**DOI:** 10.3390/jcm15051929

**Published:** 2026-03-03

**Authors:** Siddharth Shukla, Prachet Balaji, Ilayda Ozsan McMillan, Marvyn R. Arévalo Avalos, Harpreet Nagra, Zara Dana

**Affiliations:** Supportiv, 2222 Harold Way, Berkeley, CA 94704, USA; siddharth@supportiv.com (S.S.); prachet@supportiv.com (P.B.); ilayda@supportiv.com (I.O.M.); marvyn@supportiv.com (M.R.A.A.); zara@supportiv.com (Z.D.)

**Keywords:** suicidal ideation, digital peer support, artificial intelligence, machine learning, crisis, depression, distress

## Abstract

**Background**: Suicidality continues to rise, while mental health services face obstacles of access, availability, and affordability. Digital peer support (DPS) may help bridge these gaps and facilitate early identification of suicidal ideation (SI). **Objective**: This study examined (1) the effectiveness of a hybrid solution combining a proprietary AI-based SI detection with real-time human moderation within DPS, (2) distribution of SI, (3) active SI referral, (4) linguistic differences in SI, (5) sentiment changes among users, and (6) the effects of peer SI disclosure. **Methods**: We retrospectively analyzed 169,181 live-chat transcripts encompassing 449,946 user visits (January–December 2024) from a DPS provider, Supportiv. Passive and active SI were identified using a hybrid AI and human moderator solution with post hoc LLM verification. Sentiment analysis and ANCOVA compared changes in sentiment across three propensity-matched user groups: passive SI users, non-SI users exposed to peer SI, and non-SI users not exposed to SI. **Results**: SI occurred in 3.19% of live chats. The AI model identified SI faster than humans (in 77.52% passive and 81.26% active cases), with 90.3% agreement. Moderators followed up 71.3 s after AI alerts and referred 5472 active SI users (1.21%) to crisis care. All users significantly benefited from DPS, with reductions up to 29.3% in depression, 26.8% in loneliness, 25.3% in despair, and 22.3% in helplessness, with optimism increasing up to 40.4%. **Conclusions**: AI-integrated, human-moderated DPS offers scalable and effective support for high-risk populations. The proprietary SI detection AI model accurately detects suicidality, allowing for human-moderated DPS to improve the mental well-being of users with and without SI, and maintains peer safety.

## 1. Introduction

Suicide rates are on the rise in the U.S. and worldwide [1,2]. According to the CDC, in 2023, 12.8 million people in the U.S. seriously considered suicide, 3.7 million made a plan, and 1.5 million attempted it [3]. It is also estimated that over 720,000 deaths are attributed to suicide annually, and it is the third most common cause of death for people aged 15–29 [1]. Mental health services are not able to meet the demand for this urgent and growing need for care [4,5]. As of 2024, approximately 122 million Americans live in areas lacking sufficient mental health services [6], and many individuals diagnosed with a serious mental illness have not received treatment [7]. With the rise of novel and innovative approaches, digital peer support may offer an alternative pathway to care for general mental health concerns and an accessible intervention for suicidality.

### 1.1. Suicidality and Emotional Health

Suicidality encompasses the risk of a suicide attempt, characterized by suicidal ideation (SI), intent, and active planning. SI is often the initial trigger that can lead to the development of intent and planning, making early detection of SI critical in suicide-prevention efforts [8]. SI may develop passively with thoughts of death and not wanting to live anymore without a concrete plan (passive SI), whereas the presence of a specific plan for when and how one will commit suicide defines active SI [9]. While passive SI is more common, it is often overlooked and can develop into active SI when left unaddressed [10,11]. Active SI is less common but carries imminent danger, with approximately 10% of individuals with active SI attempting suicide, often within a year of developing active SI [12].

Key emotions associated with suicidality include despair, helplessness, loneliness, and depression, while optimism is protective [13,14,15,16,17]. Despair and helplessness are manifested by a perceived lack of control and are linked to increased SI risk [16,18]. Loneliness also predicts higher SI risk across cultures, genders, and age groups [17,19,20]. Depression is one of the most established risk factors for suicidality, especially in teens and young adults, highlighting the need for mental healthcare that resonates with the younger population [21,22].

Depression also interacts with other risk factors for suicidality, like helplessness and loneliness, altogether increasing SI and the likelihood of transitioning to suicidal attempts [23,24,25,26,27]. Considering the strong link between depression and suicidality, it is critical to identify and address depression promptly. In fact, treating depression in a timely manner can decrease suicide risk by 30% [28]. In contrast, optimism promotes resilience and lowers SI risk by enhancing coping, resilience, and problem-solving behaviors [29,30,31,32]. Because these emotions related to SI risk can be identified early, prevention efforts may aid in reducing suicide attempts. However, access to typical mental health services remains limited, creating the need for innovative and scalable solutions, including digital peer support.

### 1.2. Digital Peer Support

Peer support offers greater accessibility and a sense of community compared to typical mental health services. Evidence from extensive reviews showed that peer support delivered through multiple formats, including in-person, phone, text, and online support, can reduce SI [33], increase hopefulness [34] and social support, and reduce suicidal behaviors [35], enhancing belonging and reducing hopelessness [33], which are two mechanisms that lead to SI according to the Interpersonal Theory of Suicide [36]. Peer support also reduces depression [37,38] and positively impacts optimism, hopefulness, coping, and resilience [32,33,34]. Though typical peer support has shown positive outcomes in suicide prevention, the impact and mechanisms of digital peer support (DPS) on suicidality prevention is unclear.

Digital mental health services, such as mobile applications, web-based chats, and crisis text lines, have been shown to effectively aid individuals with common mental health concerns, including SI [39,40,41]. Such services effectively reduce anxiety, depression, and SI by overcoming barriers to care, such as stigma, cost, and limited access and availability [42,43,44]. An innovative digital tool is digital peer support (DPS), which is a type of online peer-to-peer support service, and it has recently gained significant traction as an effective and accessible solution to assist individuals with emotional struggles [45,46,47]. DPS services can be one-on-one or in a group setting, synchronously or asynchronously, and may be unmoderated or moderated. Moderation denotes synchronous, interactive oversight, and it should not be conflated with after-the-fact content adjustment [48]. DPS programs may also be anonymous or authenticated, a wiki-post/forum chat, SMS-based, live chat-based, video-based, or audio-based [49,50]. Current research indicates that DPS can effectively reduce feelings of loneliness, sadness, insecurity, stress, and anxiety, and promote emotional resilience [51,52]. Its accessibility, anonymity, and community-based structure can further reduce stigma and encourage help-seeking among individuals that are hesitant to seek professional help [53,54,55]. While peer support and DPS are not replacements for emergency in-person services, they may play a pivotal role in early detection and response to mental health crises, emotional stabilization, and referral to appropriate care. In crisis contexts like SI, real-time moderation is particularly important to ensure user safety by enforcing protective rules that promote a positive and safe environment [56]. Understanding DPS’s impact on conversations that escalate to crisis situations is essential for developing effective digital suicide prevention interventions.

### 1.3. Hybrid Artificial Intelligence and Human Moderation

Artificial intelligence (AI) language models can detect direct expression of SI text more rapidly than humans [57,58,59,60,61,62], but AI often struggles with nuance, figurative language, and contextual understanding, which may produce false negatives for SI [63,64,65]. For example, for a person in distress, disclosing SI may be uncomfortable to label directly, leading individuals to frequently use idioms, colloquialisms, slang, jokes, and metaphors with conversational undertones to express their emotions [66]. In many instances, AI chatbots fail to recognize true SI, and they also enable dangerous behavior by providing information on harmful methods and inducing psychosis about the user’s self, family, and surroundings [67,68,69,70,71,72]. Additionally, the AI chatbots fail to provide resources or appropriate support. One study found that resources and crisis support was provided in only an approximate third of instances deemed necessary, compared to the less-than-10% failure rate of licensed professionals [73].

Furthermore, these general-purpose LLM chatbots, such as ChatGPT, Claude, or Gemini, are not designed as safety systems [74]. They rely on user-initiated interactions; are malleable in their responses based on user instructions; and rely on simple safety policies without user history, context, or any real-time human oversight. While humans can engage in conversations without utter agreeableness, as well as subtly challenge negative thoughts, LLM chatbots over-agree in an attempt to validate the user [75,76]. In fact, recent studies suggest that LLMs on their own have a tendency to cause or perpetuate user delusions and worsen SI [73,77]. Due to this over-agreement, users may also experience feeling unheard, invalidated, and further agitated [78]. Approximately 1.2 million users weekly discuss SI on ChatGPT alone [79]. While AI can detect direct SI language quicker than humans, AI still struggles to recognize subtler signs, over-agrees with the user’s thinking, and struggles to subtly challenge negative or dangerous ideation in a productive and ethical manner. Therefore, AI therapy chatbots are not effective solutions on their own for detecting and responding to crisis situations, as they frequently hallucinate, provide dangerous advice, and miss critical cues to impending user crises [80]. Moreover, the training basis for LLMs are not publicly available, evading systematic evaluation, and most AI chatbots are designed for Western-centric content, reflecting cultural bias.

As AI-supported digital mental health tools are becoming increasingly common, there are concerns relating to model transparency, and evidence of safety and effectiveness [81]. Many models employing machine learning in the healthcare-related field do not offer insight into how output is generated, posing challenges in important contexts where decisions carry critical implications, such as in assessing and reacting to suicidality [82]. Explainable AI is essential for promoting trust, supporting human oversight, and assisting decision-making, especially in high-stakes mental healthcare settings [79]. These factors highlight the importance of human and AI hybrid solutions [83]. DPS services can offer human oversight and predefined safety structures, while employing similar AI LLMs as supportive rather than sole measures of SI detection and protection. This hybrid approach allows for more effective crisis identification and intervention.

In its current state, AI is more scalable, whereas humans are better equipped to recognize subtle nuances and respond appropriately to a person’s mental health needs [84]. In fact, a 2025 study found that over 6000 participants found “human-attributed” responses more supportive and emotionally resonant than AI-generated responses, and they were willing to wait days to receive human-generated feedback [85]. Despite simulating empathy, AI models cannot feel it, and the human touch remains irreplaceable and non-replicable [86].

Supportiv’s DPS solution leverages AI to facilitate these real human connections, rather than trying to mimic them with a bot. Therefore, in a digital, live chat-based environment, AI may conduct textual analysis to flag potential at-risk individuals, while human moderators provide simultaneous real-time oversight to assess user sentiment and respond appropriately to their needs by conversing with them or connecting them with crisis lines when needed [87]. However, it is not clear how human moderators and peer interactions jointly influence outcomes in crisis scenarios, including SI detection. Further, additional research is needed to determine the effectiveness of moderators in helping live chat-based and peer supported users with SI [88].

### 1.4. The Present Study

The objectives of this research are to (1) assess the effectiveness of a hybrid SI detection solution in a DPS setting, Supportiv; (2) measure timeliness of referrals to higher-level crisis care for users disclosing active SI; (3) characterize linguistic distinctions between passive and active SI disclosures; (4) examine DPS’s impact on emotional health of users with passive SI and non-SI users; and (5) investigate the influence of peer SI disclosures on the emotional trajectories of exposed versus non-exposed users. Together, these objectives focus on a DPS service’s capacity to detect and respond to SI in a timely manner, to elucidate both individual and group-level interactions during moments of SI disclosure, and to evaluate an AI-supported and human moderated hybrid solution in shaping emotional outcomes among DPS users.

## 2. Materials and Methods

### 2.1. Supportiv Service Solution

Supportiv (Berkeley, CA, USA) is a U.S.-based, anonymous, 24/7 DPS service providing mental, emotional, and social support through real-time moderated, synchronous small-group live chats. Access to the privately owned service is offered through employer benefits programs, medical plans, and government health plans or can be purchased directly by an individual. Users begin each session with the prompt, “What’s your struggle?” And then they share a free-text description of their present concern. Using AI-driven Natural Language Processing (NLP), users are matched with up to four other peers facing similar issues. Each synchronous small-group live chat is facilitated by a trained human moderator, supported by AI models that detect emotional expressions and flag potential suicidality.

Moderators ensure a psychologically safe environment in real time by enforcing live-chat rules, facilitating empathetic conversation, leading problem-solving efforts, and intervening when needed, especially during moments of crisis, when protocols must be enacted to ensure prompt referrals to higher-acuity crisis care for users in need. They have completed formal sub-clinical training and have demonstrated competence in skills such as asking facilitative questions, providing an empathetic experience, sharing relevant resources, and, when necessary, making service referrals. Moderators are continuously supervised for their handling of crises and regularly monitored on all aspects of service delivery quality.

The service collects no personally identifying information (PII), personal health information (PHI), or HIPAA-protected health information, and demographic data are voluntarily provided by the users at the end of the sessions. Prior to accessing services, individuals are required to review and consent to the Terms of Use and Privacy Policy, including active consent for the use of their anonymous data, which are inherently anonymous from the outset, not post-collection, for research and service-improvement purposes. Supportiv complies with General Data Protection Regulation (GDPR) and California Consumer Privacy Act (CCPA) laws.

All authors are current or former employees of Supportiv. Analyses were performed on anonymous user data following predefined study protocols to mitigate any sources of bias. Author affiliation is disclosed for transparency and does not influence the outcomes.

### 2.2. Study Design and User Classification

We conducted a retrospective observational analysis of 169,181 live-chat transcripts from 1 January to 31 December 2024, representing 449,946 user visits from 165,149 unique individuals. Only chat sessions containing at least three messages from a user were included in the analysis. This inclusion criterion was applied to confirm there was enough conversational depth for linguistic and sentiment analyses, which are not possible in brief interactions. Users were grouped into three categories: (1) users disclosing suicidality (including subgroups of users classified as disclosing active or passive SI), (2) non-suicidal users exposed to SI disclosures, and (3) non-suicidal users not exposed to suicidality disclosures. This study was approved by Pearl IRB via IRB ID 2026-0020 on 15 January 2026.

### 2.3. Identification and Verification of Suicidality

The identification of passive and active SI involved a hybrid AI model and human moderation solution with a multi-step protocol. To help moderators identify these situations, we have developed a proprietary AI crisis detection model that works as a human-supervised decision-support tool [89]. Using patterns found in thousands of manually labeled messages, the 125M-parameter RoBERTa-based binary classifier model trained on a dataset of peer support chat messages that were manually selected by experts [90]. All messages were anonymous at the point of generation, rather than anonymized post hoc, reducing privacy risk and preserving linguistic expression profiles. An active learning approach prioritized examples with high uncertainty or proximity to the model’s decision boundary to improve its performance. The expert-labeled examples were used to fine-tune the model’s performance in areas with the highest misclassification risk. Model outputs were continuously monitored for false positives and false negatives. Identified errors were followed by retraining, which allowed the model to evolve linguistic detection. This continuous refinement process supports internal validity and improves generalizability across different users and expression styles. Since conversations frequently included indirect, metaphorical, or sarcastic language, trained human moderators provided oversight to the AI model, especially when a nuanced understanding was needed. This moderator feedback also further refined the model.

The AI model rapidly analyzed user messages to identify linguistic features, sentiment-related cues, and contextual patterns indicative of crisis risk (Appendix A). When flagged, moderators made rapid and effective judgments to initiate a standardized SI risk assessment protocol, based on questions from the Columbia-Suicide Severity Rating Scale [91], by asking a user, “Do you want to kill yourself right now?” Users who denied intent but discussed suicidality were classified as passive SI. Affirmative answers or inability to confirm safety produced an immediate crisis-referral protocol, classifying the user with active SI. In this protocol, the moderator expressed concern for the user’s safety and informed them of the need for additional support. This referral was implemented through an automatic screen re-direct that presented a list of one-click live crisis resources on the next chat (e.g., National Suicide Prevention Hotline and 911). Moderators could also initiate the protocol based on their judgment and as many times as necessary within a single chat session. This solution facilitated accurate and timely identification of SI, ensuring that at-risk users receive the appropriate resources.

To assess the reliability of the AI model, we employed an additional verification layer using an LLM (GPT-4o mini). Discrepancies between the LLM classifications and moderator judgment were rare and were reviewed by a clinical expert. Final classifications were based on moderator judgment. We additionally examined linguistic differences between active SI and passive SI using n-gram analysis, monthly SI distribution, and associations with user-reported demographic data.

### 2.4. N-Gram Linguistic Analysis

To identify differences in linguistic patterns analogous to the differences observed in their emotion trajectories between active and passive SI users, we conducted the n-gram frequency analysis. All sessions were embedded via a pretrained sentence transformer AI model (all-MiniLM-L6-v2). For every message within a session, 2 to 5 candidate phrases were ranked using cosine similarity to these embeddings, filtered for duplicates, and frequency sorted. This produced 199,786 messages from passive SI users, and 69,408 messages from active SI users, with passive SI contributing 6256 phrases (frequency > 1) and active SI contributing 1606 phrases to the final grouping.

### 2.5. Sentiment Scoring and Emotional Outcomes

We examined emotional trajectories for despair, loneliness, helplessness, depression, and optimism. These emotions were chosen due to their strong association with suicidality and clinical distress, as established in the psychological literature [15,16,17,19,30,32]. Each user message was scored on a 1–10 intensity scale by a GPT-4o-mini model, employing a few-shot learning approach with representative labeled examples [89]. This emotion scoring method, including scoring criteria, was previously validated against human ratings and demonstrated robust consistency across different emotions [89]. Prior research has demonstrated that this approach is comparable to or superior to State-of-the-Art models [92]. This study does not introduce a novel scoring method; instead, it applies an established framework. Messages with insufficient emotional inference content, such as unrelated statements, were excluded from scoring. Examples for the scores 1, 5, and 10 are provided in Appendix B.

### 2.6. Emotional Outcome Analysis

To assess changes in emotional state over the course of a DPS chat, we analyzed emotion scores continuously as the conversation progressed. Conversations were mapped onto a 0 to 100% progression scale using linear interpolation so that chats of differing lengths could be compared on a common timeline. Normalizing the timeline allowed for aggregate population-level analysis of conversations that naturally varied in length. While emotional change, especially during crisis support, is not linear and may involve momentary improvements and deteriorations, linear interpolation presents an opportunity to compare conversations at scale.

Users that were classified as exhibiting active SI were excluded from emotional trajectory analyses, as an active suicidal ideation flag immediately triggered a crisis referral protocol and this referral altered the structure and goals of the DPS interactions. Instead, users with active SI were analyzed based on the hybrid solution’s detection and referral timelines.

To assess the impact of the DPS on the emotional health of users disclosing SI or exposed to SI disclosures, we restricted analyses to chats that began with measurable negative emotion. For each conversation, we reviewed the first three user messages and calculated the maximum score for all four negative emotions. Conversations were included for a given emotion if at least one of these first three messages scored ≥ 5 on a 1–10 scale. This approach accounted for the possibility that initial messages might contain greetings or introductions before transitioning to the core issue, which typically reflects the emotional state of concern. Since this thresholding method was applied separately for each emotion, different subsets of conversations were used depending on the emotional outcome. This ensured trajectories reflected emotional distress and allowed a targeted assessment of how peer support influenced emotional trajectories throughout the conversation. Conversation topics were assigned using LLM-based categorization into predefined topics. Topics were validated manually and used for propensity matching to ensure balanced comparisons between user groups. Propensity scores were estimated using logistic regression with two covariates: (1) initial emotional intensity, defined as the maximum emotion score within the first three messages of each conversation, and (2) conversation topic, included as a categorical variable with one-hot encoding. Propensity scores were transformed to the logit scale to improve distributional properties for matching. One-to-one nearest-neighbor matching without replacement was performed, pairing each treatment group conversation with its closest control group conversation based on logit-transformed propensity scores. A caliper of 0.01 on the logit scale was applied to ensure close propensity-score proximity between matched pairs. Pairs exceeding this threshold were excluded from subsequent analyses. The tight caliper restriction ensures that matched pairs have near-identical propensity scores, indicating comparable distributions of the matching covariates between groups.

#### 2.6.1. Group Comparisons

To evaluate the effectiveness of DPS in supporting users who disclosed SI, as well as non-suicidal users exposed to peer SI, we constructed two separate analytic scenarios comparing users’ emotional sentiment trajectories over the course of a single live chat. The first comparison evaluates emotional trajectories of users disclosing passive SI compared with non-suicidal users who were not exposed to crisis-related content or SI disclosures (non-SI). This comparison tests whether individuals expressing passive SI exhibit different emotional trajectories than those seeking general emotional support. The second comparison investigates the emotional trajectories of users exposed to peer SI disclosures. The goal is to assess whether the emotional states of exposed peers are significantly affected by the presence of suicidal language within a shared digital space. This analysis offers insight into the indirect psychological impact of crisis exposure on vulnerable users. In every analysis, the control group consisted of matched users from regular chats. Matching was based on initial emotional intensity and conversation topic.

#### 2.6.2. Statistical Analyses

Baseline emotional scores were operationalized from each user’s first message within a session, after filtering for users who contributed at least five messages per room. Zero-value scores (indicating no emotion detected) were excluded from the analysis. Group differences in baseline emotional scores were assessed using the Mann–Whitney U test, a non-parametric alternative appropriate for ordinal rating scale. Using Analysis of Covariance (ANCOVA), we determined whether users’ emotional trajectories differed across groups. The independent variable was group (e.g., passively suicidal vs. non-SI user), while the covariate was normalized message progression across the conversation. The dependent variables were emotion scores, with the key term of interest being the interaction between group and time to indicate whether emotional improvement over time differed between user groups.

## 3. Results

Between 1 January and 31 December 2024, Supportiv hosted 169,181 peer-to-peer support live chats. Among these, 8929 (1.98%) were classified as containing passive SI, while 5472 (1.21%) involved active SI, meaning that 3.19% of all sessions included SI disclosure. Across the same period, DPS live-chat sessions received 449,946 total user visits from 165,149 unique individuals. In total, 22,346 user visits, representing some repeat individuals from different sessions, occurred in chats where a user disclosed SI. Overall, 4.97% of users either disclosed SI themselves or were exposed to a peer’s SI disclosure.

### 3.1. Crisis Model Evaluation for SI Detection

To assess the effectiveness of the hybrid SI detection solution, we examined the frequency and timing of AI SI-risk flags and moderator SI-risk protocol messages. The AI model is designed to assist moderators by flagging potential SI risk, but moderators also have the ability to flag risk and initiate the risk protocol even without an AI-model risk flag (though the AI SI-risk flag could be present later in the chat). In this study, we observed an overall agreement of 90.26% (true positives: 12,602; true negatives: 140,666) between the AI model and human moderators, with disagreements mostly in AI-only flags (false positives: 14,735) versus moderator-only flags (false negatives: 1799; Figure 1). The agreement rate between the AI model and human moderators was in alignment with industry standards [93].

As noted earlier, SI verification was conducted using an LLM using GPT-4o mini, and it supported the conclusion that moderators’ initiation of the risk protocol represents the actual presence of SI. Using the moderator’s assessment as the benchmark for SI risk, we calculated the sensitivity, specificity, precision, and negative predictive value of the AI-only model in detecting SI:

- Sensitivity = true positives/(true positives + false negatives) = 87.51%.
- Specificity = true negatives/(true negatives + false positives) = 90.52%.
- Precision = true positives/(true positives + false positives) = 46.10%.
- Negative predictive value = true negative/(true negatives + false negatives) = 98.74%.
- Accuracy = (true positives + true negatives)/(all total detections) = 90.26%.

The model was optimized to prioritize sensitivity over precision. In crisis circumstances such as SI detection, false negatives are markedly riskier than false positives. Therefore, sensitivity was higher (87.51%), with a lower precision rate (46.10%). This trade-off was intentional, serving as a precautionary measure. Any potentially risky language was flagged for a human review.

Collectively, these results show that the AI model performed with a high degree of accuracy (90.26%), as it correctly classified SI risk 87.51% of the time and ruled out non-SI risk 90.52% of the time. This rate was in alignment with industry standards that deemed levels above 80–85% to qualify as a high level of agreement [93]. Precision (46.10%) was relatively lower, due to a high rate of false-positive SI-risk flags that required moderators to assess for an accurate classification of SI. The high negative predictive value signaled that all of the users the model classified as not having SI truly did not have SI, thereby indicating that users with SI were not disregarded.

Additionally, we examined the AI model’s effectiveness in supporting moderators to identify SI risk and initiate the risk protocol. We examined all user chat transcripts and recorded the elapsed time from chat initiation to when the AI model flagged a message as suicidal (termed T1) and the elapsed time from chat initiation to when the moderator posed the crisis protocol question, “Do you want to kill yourself?” (termed T2). Our results showed that in 81.26% of active SI cases and in 77.52% of passive SI cases, the AI model flagged suicidal content before the human moderator (as designed), thus facilitating a quick response by the moderator. Specifically, for active SI cases, moderators initiated the risk assessment protocol 71.32 s on average (SD = 89.00 s) after the AI’s SI-risk flag. This time included recognizing the SI risk within the conversational context, typing the crisis determination questions, and raising the crisis alert when needed. Therefore, it was a more deliberate and precise assessment when compared to an instantaneous flag by the AI model.

Users identified as experiencing active SI were removed from live peer chats and referred to crisis care services. The crisis protocol involved the moderator asking “Do you want to kill yourself right now?” If the user responded affirmatively, the moderator initiated a referral to crisis care services. If the user gave an ambiguous answer, the moderator followed up with the question, “Can you commit to staying safe for the next 24 h?” Users who declined to commit to safety were also referred to crisis services. Upon referral, users were directed to a web-based crisis support page containing links and information for crisis text lines, national suicide prevention hotlines, and emergency medical services, providing immediate access to professional help.

**Figure 2:**
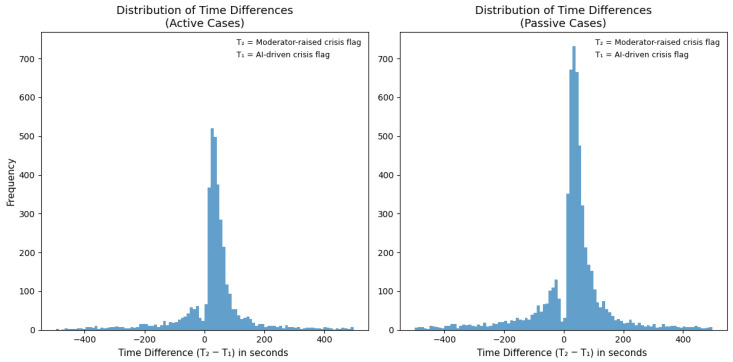
Distribution of time differences between the AI model-driven crisis flag (T1) and the moderator-raised crisis flag (T2) for SI. The *x*-axis represents the difference in time (seconds), with negative values representing cases where moderators initiated the SI risk protocol before the AI model-driven flag. The *y*-axis shows the number of cases. In most instances, the AI model detected risk before moderators enacted the SI protocol, reflecting its speed in automated detection while human moderators assessed chat context and formulated a sensitive and appropriate response.

For passive SI cases, the moderator response time following the AI’s SI-risk flag averaged 79.87 s (SD = 95.71 s; Figure 2). This time interval reflects not only detection but also the time moderators take to assess the context and compose an appropriate and sensitive response, as well as sending the correct protocol questions. While the AI model detected risk indicators very swiftly, the moderators judged human nuances in user language and context, showing sensitivity in their responses. These findings highlight how the AI model identifies SI rapidly, allowing moderators to reach out to users with a thoughtfully formulated response. In crisis conditions, the rapid detection and human sensitivity are critical for effectively managing SI risk.

### 3.2. Timeliness of Crisis Referral

In order to assess the responsiveness of the hybrid SI detection solution, we examined the time between moderators’ final crisis determination question and the delivery of crisis referral resources to users identified as high SI risk. On average, moderators shared crisis resources 245 s (SD = 557 s) after their final assessment question. The referral was not immediate, mostly because moderators and peers commonly continued interacting with the user in this time window, allowing moderators to verify risk, de-escalate, and maintain emotional validation before initiating the crisis referral process. This duration showed that moderators balanced timely sharing of crisis resources with a user and context-focused assessment, which may enhance the safety and openness of the user to the shared resources. There is no industry standard for how quickly users are referred to crisis resources following suicide-risk disclosure; therefore, the disclosed 245 s referral window provides a rare quantitative measure in digital mental health, specifically in DPS. Since the users with active SI were referred out of DPS sessions to appropriate and helpful resources to address their immediate needs, their emotional trajectories were not examined.

### 3.3. Distribution of Suicidality Cases

**Figure 3:**
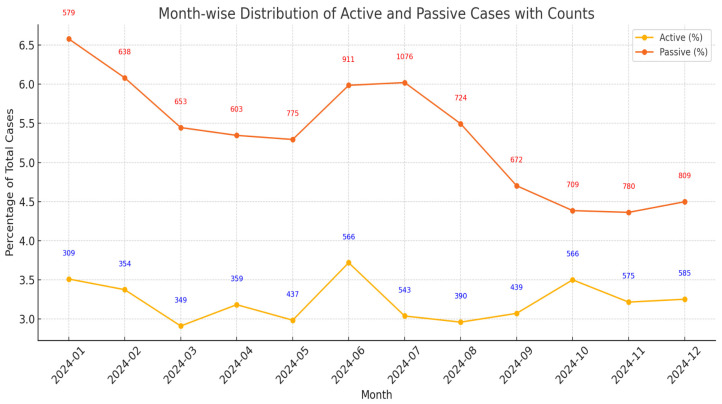
Monthly distribution of active SI and passive SI cases (%). The plotted data include cases from January 2024 to December 2024. The *y*-axis denotes the percentage of SI cases over the total DPS cases, while the numbers above the plotted points indicate the number of SI cases.

In total, there were 449,946 user visits. In all, 8929 users reported passive SI (1.98% of all users), and 5472 users reported active SI (1.21%). Passive SI consistently accounted for a higher proportion of total monthly chats than active SI, approximately at a 1.6:1 ratio (Figure 3).

Passive SI ideation peaked in July (n = 1076) and in June (n = 911), while active cases peaked in December (n = 585) and November (n = 575). The timing of these peaks may correspond to major academic transitions such as the end of academic terms and the start of summer break, the times known to exacerbate emotional stress, especially in younger individuals, as well as to winter months, which have fewer daylight hours and are associated with seasonal affective disorder [94,95]. Similarly, in passive SI, there is a slight resurgence during the end of the year. Overall, SI showed a persistent presence throughout the year despite peaking at certain months. However, when the ratio of SI disclosures to total monthly live chats were compared, users were more likely to disclose passive SI in January (accounting for ~6.6% of all chats) and active SI in June (accounting for ~3.7% of all chats).

### 3.4. Demographic Distribution of Users

Most users did not disclose their gender (69.39%). Among users that disclosed active SI, 63.10% identified as female, 27.58% as male, and 9.31% as non-binary. A similar pattern emerged in users with passive SI; 64.67% of users identified as female, 27.55% as male, 7.78% as non-binary. For users with no SI, 63.60% identified as female, 29.29% as male, and 7.11% as non-binary.

Similarly, most users did not disclose their race or ethnicity. Among users that shared their demographic information, the largest majorities identified as White (47.93% of active SI users, 48.56% of passive SI users, and 47.62% of users with non-SI), followed by Asian (15.15% of active SI, 14.09% of passive SI, and 14.53% of non-SI); Hispanic or Latino (12.09% of active SI, 13.40% of passive SI, and 13.48% of non-SI); Black or African American (11.78% of active SI,10.26% of passive SI, 9.95% of non-SI); American Indian or Alaska Native (5.98% of active SI, 6.46% of passive SI, and 7.13% of non-SI); Middle Eastern or North African (4.77% of active SI, 4.99% of passive SI, and 4.92% of non-SI); and Native Hawaiian or other Pacific Islander (2.29% of active SI, 2.25% of passive SI, 2.37% of non-SI). Overall, race and ethnicity rates were consistent across groups.

**Figure 4:**
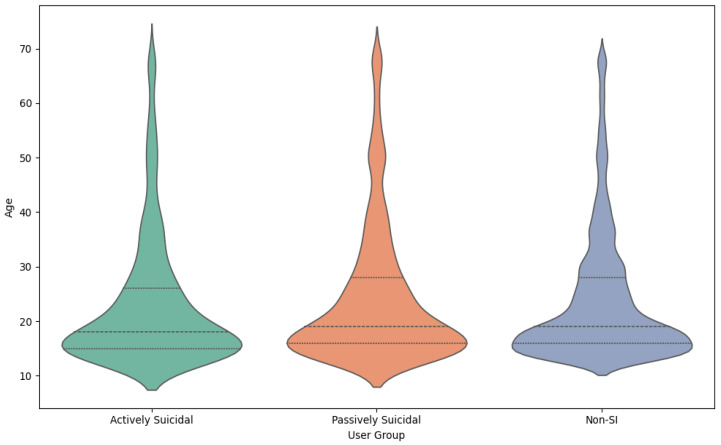
Age distribution of users (active SI, passive SI, and non-SI users). Most users of the DPS service were adolescents and young adults, as indicated by the user distribution. The wider dashed lines on the middle of the violin plots represent the median age, and the top and bottom dashed lines represent the first quartile and the third quartile.

Age data voluntarily provided by users during conversations offered further insight into the demographic profile associated with crisis disclosures. The majority of users who disclosed either passive or active SI were between the ages of 13 and 30, with the highest density falling in ages 16–22 across all groups (Figure 4). This distribution is consistent with the epidemiological data identifying adolescence and emerging adulthood as critical risk periods [96]. For users with active SI, the median age was 18 (n = 1537, mean = 23.10, and SD = 12.08); meanwhile, users reporting passive SI had a median age of 19 (n = 3343, mean = 24.42, and SD = 12.74), and users without SI had a median age of 19 (n = 40,371, mean = 23.90, and SD = 11.94). It is important to note that this demographic information was voluntarily self-reported by users in a fully anonymous environment. As a result, the demographic data likely underrepresent the full diversity of the user population and should be interpreted as indicative rather than definitive. Certain groups may be less likely to disclose demographic information; however, the consistency of observed trends provides reasonable confidence in the findings.

### 3.5. Linguistic Differences Between Active and Passive Users

We analyzed messages from individuals identified as having active and passive SI to characterize their linguistic style and determine how the two groups differ in their communicative intent and style. Table 1 illustrates that individuals with active SI used language reflecting self-harm and explicit intent to end their lives, whereas those with passive SI primarily expressed despair and hopelessness, without explicit indications of suicidal intent. Understanding these linguistic differences is advantageous for training humans and AI models to detect risk and respond much faster and recommend appropriate resources given the context. For human moderators, distinguishing between immediate risk in active SI and chronic distress in passive SI allows for adjusting strategies and taking urgent action when needed. For an AI model, incorporating these linguistic differences into its training data can refine the model’s ability to detect suicide-risk levels, reduce false negatives and positives, and improve the efficacy and safety of the DPS service.

### 3.6. Comparison of Emotional Trajectories in Passive SI and Non-SI Users

At baseline, users with passive SI indicated significantly accentuated emotional distress across all emotional outcomes when compared to users without SI (Table 2). Passive SI users displayed higher despair, loneliness, helplessness, and depression, and they had lower optimism (all *p* < 0.001). These results show that even before further participating in DPS sessions, passive SI users entered live chats with significantly worse emotional states. Notably, users were not propensity matched for scores recorded in Table 2, and the emotional scores reflect average user scores from the beginning and end of live peer chat sessions.

In all dimensions, both the users experiencing passive SI and non-SI users exhibited emotional improvement after using the DPS service based on the slopes of the emotional trajectories. When the clinically relevant dimensions of despair, loneliness, helplessness, and depression were analyzed, similar reductions after using DPS were observed in both groups, while optimism was increased at comparable rates. Passive SI users reported the highest reductions in depression by 26.3%, followed by 23.8% reduction in loneliness, 23.0% in despair, and 20.6% in helplessness, whereas non-SI users reported the highest reductions in depression, by 29.0%, followed by 25.3% in despair, 24.0% in loneliness, and 21.3% in helplessness. Optimism scores were increased by 31.8% in passive SI users and 40.4% in non-SI users.

**Figure 5:**
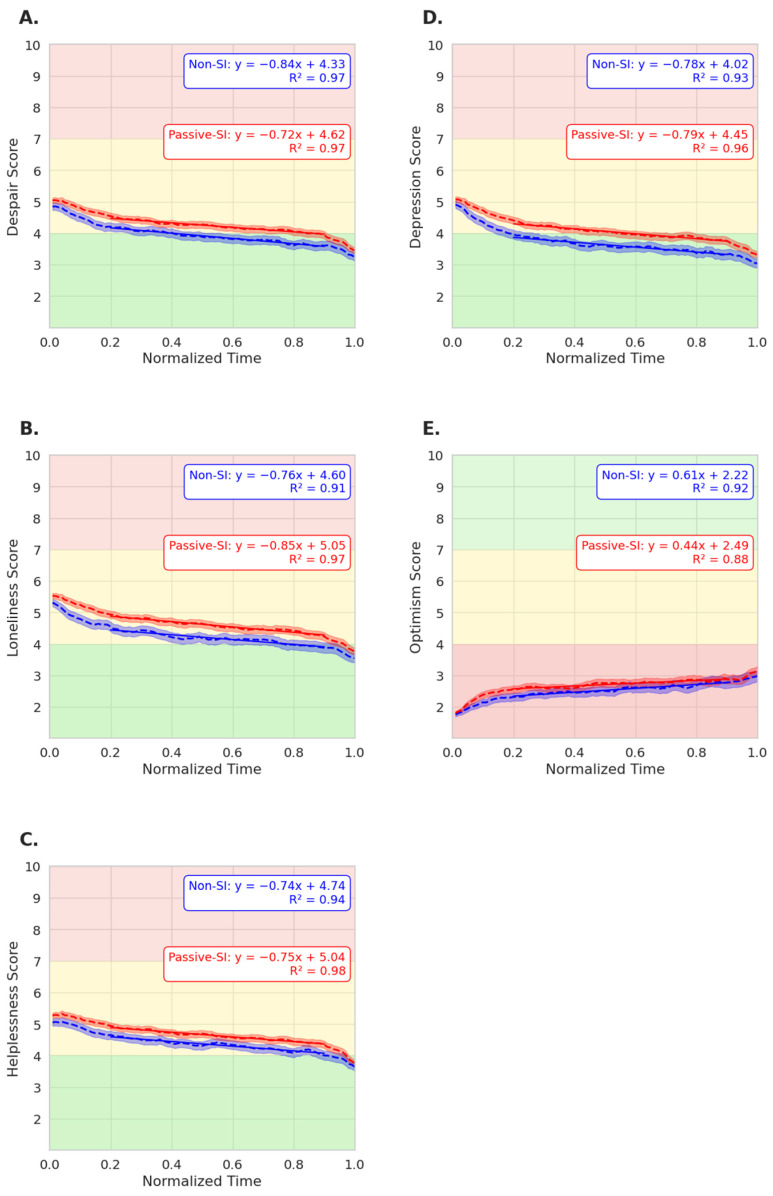
Emotional trends throughout conversation progression for users with passive SI vs. non-SI users in group sessions against time. Despair (**A**), loneliness (**B**), helplessness (**C**), depression (**D**), and optimism (**E**). The green area (1–4) represents the “low” level of emotion, yellow (4–7) signifies “moderate” levels, and red (7–10) indicates “high” emotional scores. The red lines represent non-SI users exposed to peer SI, and the blue lines represent non-SI users not exposed to peer SI.

When the trajectories of despair, loneliness, helplessness, and depression were compared throughout using the DPS service, there were no significant differences between users with and without SI; however, in optimism, a significant difference was observed. As shown in Figure 5A, the emotional trajectory of passively suicidal users in despair-eligible conversations (number of chats used for analysis: passive SI n = 4274; non-SI n = 3457) was not statistically different when compared to the emotional trajectory of non-SI users. The ANCOVA revealed no significant group-by-time interaction (*p* = 0.074), supporting the conclusion that both groups’ despair scores improved at comparable rates. Similarly, the loneliness trajectory (passive SI n = 4878; non-SI n = 4029) lacked a significant difference in slope between SI and non-SI users (*p* = 0.076; Figure 5B). Both despair and loneliness had trending *p*-values but did not reach significance. Helplessness (Figure 5C; (passive SI n = 4701; non-SI n = 3952; with ANCOVA *p* = 0.827) and depression (Figure 5D; passive SI n = 4155; non-SI n = 3410; with ANCOVA *p* = 0.248) follow the same pattern and suggest emotional benefits in users regardless of passive SI presence. However, for optimism, there was a significant group-by-time interaction (passive SI n = 2725; non-SI n =2528; *p* = 0.006; Figure 5E), suggesting higher levels of optimism increases in non-SI users. Although the emotional change over time between the groups was not statistically different for most emotions, passive suicidal users maintained slightly higher average emotional scores. However, by the end of the conversation, the two groups’ emotional scores converged.

### 3.7. Comparison of Emotional Trajectories Due to SI Exposure in Peers

All baseline negative emotional scores were significantly elevated in individuals that were exposed to peer passive SI, and optimism was lower, when compared to non-exposed users (Table 3). Much of this difference was due to users’ first messages, which assigned them to different live-chat sessions. Users who were exposed to peer SI were more likely to express greater emotional distress before joining peers that were also experiencing high distress, and SI in some cases. By contrast, the non-exposed users were placed in sessions where peers reported significantly lower emotional distress to begin with. These scores were compiled from all users without propensity matching to reflect emotional states at the beginning and end of live DPS chats.

In total, there were 22,346 user visits that were exposed to peer SI (accounting for 4.96% of all user visits). To determine whether users exposed to passive SI in shared live chats, without expressing suicidality themselves, experienced measurable emotional harm during their peer support session, we compared the emotional trajectories between exposed users and propensity score-matched users from non-crisis chats. We analyzed the following emotion categories: despair, loneliness, helplessness, depression, and optimism.

Both users exposed to SI and those not exposed reported reductions in scores across all negative emotion categories following use of DPS services. Non-SI users that were exposed to peer SI reported the highest reductions in depression, by 29.3%, followed by reductions in loneliness by 25.8%, despair by 23.7%, and helplessness of 22.3%, whereas non-SI users that were not exposed reported reductions in depression by 28.5%, loneliness by 26.8%, despair by 23.2%, and helplessness by 19.2%. Optimism was increased in both exposed (32.8%) and non-exposed (33.2%) non-SI users after DPS use.

**Figure 6:**
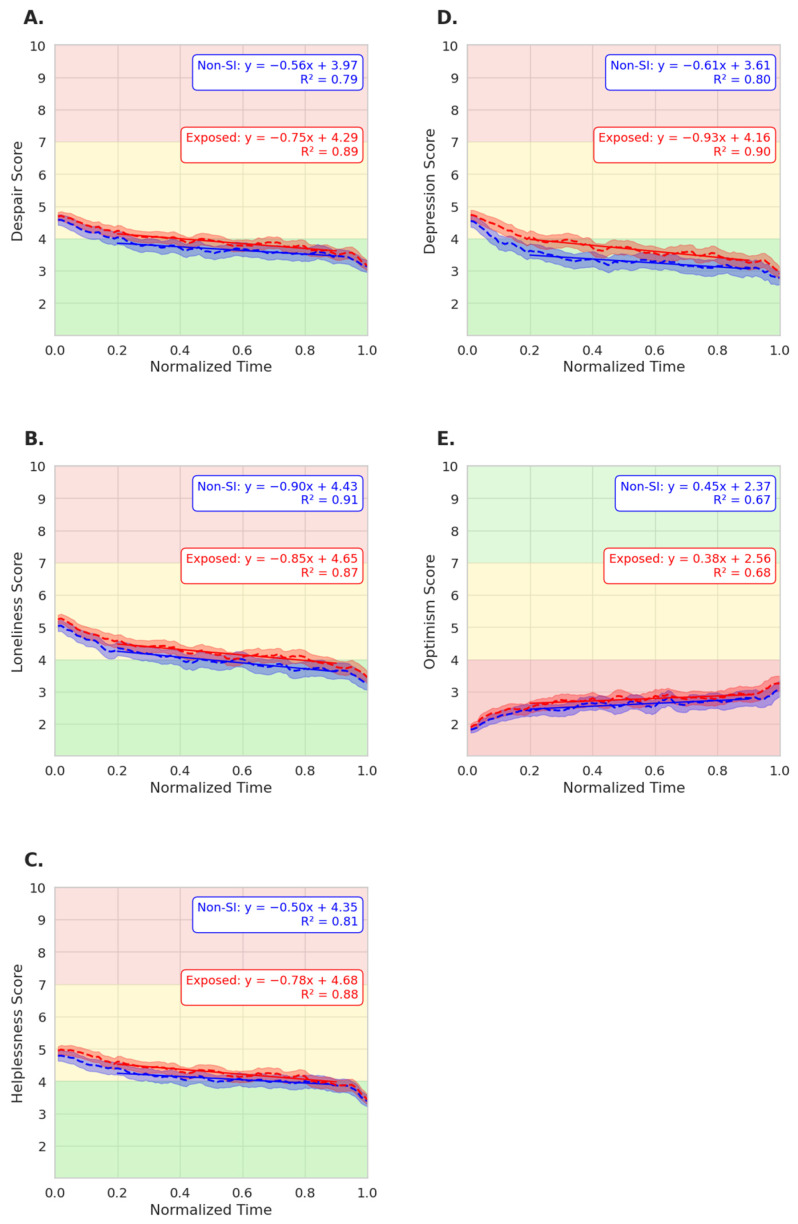
Emotional trends throughout conversation progression for users exposed to passive SI vs. non-SI users in group sessions against time. Despair (**A**), loneliness (**B**), helplessness (**C**), depression (**D**), and optimism (**E**). The green area (1–4) represents the “low” level of emotion, yellow (4–7) signifies “moderate” levels, and red (7–10) indicates “high” emotional scores. The red lines represent non-SI users exposed to peer SI, and the blue lines represent non-SI users not exposed to peer SI.

Helplessness (SI-exposed n = 1563; non-exposed n = 1411; ANCOVA *p* = 0.001; Figure 6C) and depression (SI-exposed n = 1120; non-exposed n = 989; ANCOVA *p* = 0.019) were significantly higher in users that were exposed to SI, suggesting higher initial distress in these users. In contrast, in despair scores across the conversations (SI-exposed n = 1357; non-exposed n = 1176), there was no significant group-by-time interaction (ANCOVA *p* = 0.489; Figure 6A). Similarly, for loneliness scores (SI-exposed n = 1618; non-exposed n = 1492), both groups followed parallel negative trajectories with no significant group-by-time interaction (*p* = 0.719; Figure 6B). For the protective sentiment of optimism, both peer SI-exposed and not-exposed users reported a positive trend with DPS use (SI-exposed n = 997; non-exposed n = 1022), with no significant group difference (ANCOVA *p* = 0.834). Across all the emotional sentiments analyzed, the SI-exposed group experienced higher negative affect in regards to certain emotions that were analyzed, but these scores converged by the end of the live chat.

Overall, these findings indicate that users that were exposed to SI and the users without SI benefitted similarly from DPS. Although peer SI exposure led to higher initial helplessness and depression, their emotional trajectories improved similar to non-exposed users. These results support the null hypothesis that exposure to passive SI in peers does not negatively impact the emotional state of non-suicidal users. In fact, the convergence of emotional outcomes underscores the stabilizing role of human moderators and shared empathy and structured engagement in group peer support spaces such as Supportiv.

## 4. Discussion

### 4.1. Principal Findings

DPS services, such as those offered by Supportiv, are well positioned to address unmet mental health needs [97] and improve users’ emotional outcomes via live, human-moderated peer chats. Although a growing body of evidence demonstrates the effectiveness of DPS in improving mental health outcomes [45,46,47], less was previously known about the effectiveness of a hybrid solution consisting of human moderators supported by an AI model in crisis situations, and specifically for user suicidality disclosures. The findings of this study indicate that Supportiv’s AI model effectively detects SI in real time, empowering trained sub-clinical moderators to respond in an accurate and prompt manner to these crisis situations.

Given recent advances in AI, this study examined the role of Supportiv’s proprietary AI model in relation to its ability to discern SI risk. The RoBERTa-based AI model performed with a high degree of accuracy (90.3%), correctly classifying suicide risk. These findings are aligned with a recent study in which text from the social media site Reddit was retrospectively analyzed and a RoBERTa-based model trained on these texts predicted suicide risk with a 91.5% accuracy [98]. Despite its strong performance, the AI model had a false-negative rate of 13% (i.e., did not detect when SI risk was present) and precision of 46% (i.e., only about half of the time the AI-model classified SI risk, it was correct). Comparable studies have reported false-negative rates of 2.8–8.6% [98] and precision values of 70–90% [98,99]. These discrepancies illustrate that AI models alone are not perfect and require human oversight for risk management [63,64]. Human moderators play an important role in understanding subtle nuances in language, such as sarcasm, figurative speech, humor, or idioms that may conceal user emotions or messages, as well as abbreviated or misspelled text, especially important in instances that require the timely detection of SI risk [100]. For example, AI models can be oblivious to repetitive joking about suicidality, cultural references, or linguistic tone, whereas a trained human moderator can discern the underlying message. These limitations of AI or human moderation alone highlight the need for a hybrid system for the timely detection and easier scalability of the AI, integrated with the empathy and contextual understanding of humans, for effective detection and intervention.

The AI model raised SI flags with an average of 70 s before human moderators initiated the crisis protocol. This difference is most likely due to the additional time moderators spend to recognize risk within the live-chat context, formulate a response, and initiate the required and predetermined crisis protocol steps, rather than a delay in detection. Therefore, the timing difference underscores the complementary roles of the AI model and human moderators’ judgment, maintaining speed and empathy in crisis care. Users with active SI were referred to crisis resources approximately 4 min after the moderators asked their final crisis protocol question. The referral was not immediate, as users had delayed responses in most cases possibly due to increased emotional processing, and further conversed with their peers and moderators. Overall, users with high suicidality risk were referred to helpful resources in a timely manner, while moderators maintained a balance between emotionally validating their concerns, ensuring their safety, and confirming active SI.

Importantly, this study also demonstrated that both users disclosing passive SI and non-SI users exposed to peer SI benefit emotionally from using a DPS service like Supportiv. Users experiencing active SI were not included in emotional trajectory analyses, as these cases required immediate escalation to crisis resources by moderators in order to prioritize their safety. Despite entering live chats with higher baseline levels of despair, loneliness, helplessness, and depression, as well as lower optimism, compared to non-SI users, the passive SI users’ emotional sentiments improved at similar rates compared to their non-SI peers, and outcomes were comparable across groups by the end of sessions. These results suggest that DPS is an accessible and effective option for individuals experiencing passive SI. As noted earlier, the peer-based environment facilitates social connectedness, thereby reducing loneliness and improving feelings of hopelessness (e.g., despair) and depression, which are significant predictors of suicidal ideation [35,36]. For despair, loneliness, helplessness, and depression, both users with passive SI and non-SI benefitted at similar rates from DPS use. However, a significance in group-by-time interaction in optimism scores was observed, indicating that optimism increased at a higher rate in users without passive SI, possibly due to users with SI being less likely to sustain higher levels of optimism throughout DPS participation. Given that optimism and resilience often develop through longer-term reinforcement, these effects may not be detected within the short-term scope of this study. Users dealing with severe emotional distress can achieve a similar emotional regulation as those with moderate-to-low emotional distress within the same timeframe of using this service. This aligns with prior research showing that expressing negative emotions and experiences in a supportive environment facilitates emotional processing and relief [60], and that interpersonal sharing can help regulate emotions [61]. Moreover, emerging evidence indicates that DPS can provide benefits even for individuals with a serious mental illness or significant emotional distress [62].

Subsequent analyses revealed that non-SI users who were exposed to peer SI also benefited from the DPS service. This was important to evaluate, as prior research suggested the possible social contagion of SI through unmoderated social media [63]. Across five emotional domains, both non-SI users in crisis chats (exposed to peer passive SI) and non-crisis chats (no exposure) showed significant improvements. Despite the overall benefit users derived from DPS, users indicated helplessness and depression scores that showed significant group-by-time differences over the course of sessions. No significant time-by-group differences were found for despair, loneliness, or optimism. Users reported greater emotional well-being after using the service, regardless of their exposure to passive SI.

Together, these findings suggest that engaging in a DPS environment is beneficial even when peers express high emotional distress or SI. This is encouraging evidence that crisis prevention can occur in a digital group setting without distressing the rest of the group. These results point to the value of exploring collective solutions to suicide prevention, alongside individual or more typical mental healthcare methods. Moreover, the integration of an AI model and synchronous human moderation improves services’ ability to detect and address SI risk quickly and effectively.

### 4.2. Limitations and Future Directions

These findings are encouraging for helping users at risk for SI and ensuring the safety of non-suicidal users exposed to SI; however, there are a few limitations to consider. First, the measured emotional trajectories reflect only short-term and in-session changes; therefore, they do not illustrate long-term outcomes. It is unknown how long emotional relief lasts after the users exit the service. Thus, future research focusing on the effects of similar services should examine the long-term well-being of users, possibly through subsequent chat visits or follow-up surveys.

While propensity score matching was used, unknown confounding variables may have affected the results. Some of these could include the absence of demographic information, concurrent mental health support, and individuals’ openness to share accurate emotional well-being. In an anonymous service like Supportiv, it is unfortunately impossible to gather or ensure the accuracy of all of these variables. Since sociodemographic analyses were based only on users that voluntarily disclosed personal information, there may be a selection bias if those individuals differ systematically from users that did not disclose their personal information. Approximately 30% of users disclosed demographic information, yielding a meaningful subsample to explore demographic patterns. However, these results should be interpreted with caution, as they may not be representative of a broader user population. High rates of non-disclosure may indicate preferences for privacy and stigma concerns, which may be related to the characteristics of user populations. Therefore, demographic patterns are intended to provide insights into engagement for users that willingly disclosed demographic information, rather than generalizing to a population level.

Additionally, the service context of Supportiv may influence the generalizability of results. Supportiv is a U.S.-based privately owned service that is primarily offered through employer benefits programs, medical plans, and government health plans, in addition to individual purchases. Therefore, there may be cultural, socioeconomic, and healthcare system differences that limit the generalizability of these results to non-U.S. and non-Western populations. The user pool also introduces a selection bias. Studied users may have higher motivation to seek help or a greater aptitude in digital literacy, as they were self-motivated to use DPS services. These factors may affect how users engage with DPS, in addition to how they may benefit from using such services.

Supportiv’s hybrid solution appears effective; however, the present study does not examine the AI model and human moderation separately in terms of detecting users’ SI or determining their emotional benefits from DPS use. Since the hybrid solution is fully integrated in Supportiv, it is not possible to examine each individually and deduce whether the observed emotional improvements in emotional regulation were due to human moderators, AI detection, or a combination. Future research examining services separately can clarify these effects.

It is important to note that all authors are current or former employees of Supportiv. This affiliation is reported for transparency. To mitigate any risk of introducing bias in analyzing or reporting findings, analyses were performed using anonymous data following predefined protocols. Classification of suicidality was determined by trained moderators according to standardized procedures rather than author judgement. Independent validation would further strengthen these findings.

From a broader digital health perspective, AI is most effective when it supports specific functions within a healthcare system, such as detection, triage, or decision support, while human oversight is preferred for interpretation, ethical judgement, and delivery of care [101]. DPS services that employ a hybrid solution integrate AI models that support real-time detection of SI risk, and human moderators that interpret and validate AI models’ findings, provide empathy to users, and finalize decision-making. This configuration differs from fully automated healthcare settings that are limited [102], instead offering scalability and safety as an AI-integrated mental healthcare tool. Future research should focus on how hybrid AI and human systems can be further optimized across digital health settings, workflows, thresholds for crisis escalation, and long-term outcomes.

## 5. Conclusions

This study provides evidence that a hybrid approach combining AI model-supported detection with human moderation can effectively identify and respond to suicidality. This model leverages the efficiency and consistency of AI LLMs for rapid detection of SI, while utilizing the sensitivity and judgement of the human moderators. Essentially, this solution is a “best of both worlds” framework in which the AI model can rapidly identify emotional distress and crises, and then trained moderators can provide human empathy and awareness to allow for emotional regulation. Prior research had noted the limitations of AI in comprehending emotional nuances and social context [64], as well as the challenges humans have in maintaining scalability and consistency [50,57]. Furthermore, humans and AI are able to complement each other while filling in the gaps in the other’s weak points.

Importantly, DPS use was associated with significant emotional benefits among users, especially in those disclosing passive SI, as well as among users that were exposed to peer SI. Together, these findings suggest that moderated DPS can support passively suicidal users while preventing negative effects on non-SI peers, addressing concerns relating to social contagion in online settings. Similar hybrid models can be applied not only in DPS services, but also more widely in other mental health services.

Altogether, this study highlights AI and human hybrid solutions as a scalable, efficient, and safe option for suicide prevention and emotional support for digital mental health services. As the field of digital care advances, key areas of exploration include optimizing the partnership between AI and humans and examining the potential long-term benefits for users to complement typical mental health systems and increase access to support.

## Figures and Tables

**Figure 1:**
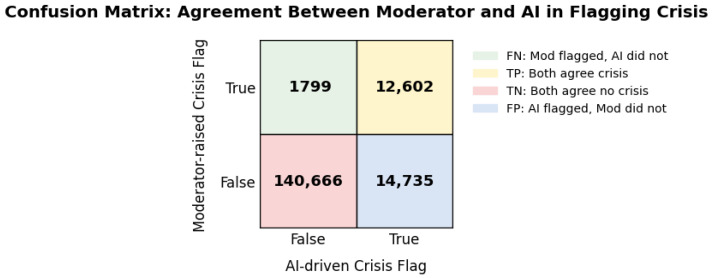
Detection of SI by the hybrid AI model and human moderator solution. “AI-driven Crisis Flag” refers to the AI suicidality flagged by the AI model. “Moderator-Raised Crisis Flag” indicates the number of times a moderator initiated the risk protocol after recognizing SI. There were 12,602 cases of true positives (TPs) that both the moderator and AI model raised crisis flags for; 1799 cases where the moderator raised a flag but the AI model did not (false negatives, FNs); 14,735 cases where AI model raised a crisis flag but the moderator did not (false positives, FPs); and 140,666 cases that both the AI model and moderator agreed not to flag (true negatives, TNs).

**Table 1:** (**A**) Top phrases used by users categorized as active SI, determined by n-gram frequency analyses. Linguistic analyses indicate that users exhibiting active SI express urgent threat to end their life. (**B**) Top phrases used by users categorized as passive SI, determined by n-gram frequency analyses. Linguistic analyses indicate that users exhibiting passive SI express feelings of unhappiness, loneliness, anxiety, and wanting to escape their current situation without any clear planning.

**(A)**	
**Active Risk N-Gram**	**Frequency**
Just wanna die	261
Feel like dying	135
Don’t want life	91
Want kill self	70
feel like failure	64
I’m better dead	29
Want end life	48
**(B)**	
**Passive Risk N-Gram**	**Frequency**
Just feel lonely	428
Just wanna disappear	193
Feel like running away	186
Just feel worthless	151
I’m really scared	137
Just hate life	122
Think autism social anxiety	83
I’m said I’m ugly	69

**Table 2:** Baseline emotional scores of users with passive SI and users without SI. Beginning and ending emotional scores are denoted, separated by a forward slash, “/.”

	Despair	Loneliness	Helplessness	Depression	Optimism
Passive SI	6.82/3.45 (SD = 1.39/2.26)	7.14/4.34 (SD = 1.40/2.32)	7.02/4.20 (SD = 1.40/2.28)	6.74/3.74 (SD = 1.33/2.19)	2.12/4.09 (SD = 0.57/2.28)
Non-SI	6.34/3.17 (SD = 1.23/1.99)	6.60/4.00 (SD = 1.26/2.11)	6.48/3.87 (SD = 1.22/2.06)	6.23/3.29 (SD = 1.17/1.92)	2.17/4.02 (SD = 0.59/2.23)
Differences between groups (*p*-value)	*p* < 0.001	*p* < 0.001	*p* < 0.001	*p* < 0.001	*p* < 0.001

**Table 3:** Baseline emotional scores in users exposed to passive SI and users that were not exposed to SI. Beginning and ending emotional scores are denoted, separated by a forward slash, “/.”

	Despair	Loneliness	Helplessness	Depression	Optimism
Exposed to passive SI	6.63/3.15 (SD = 1.38/2.05)	7.02/4.02 (SD = 1.40/2.15)	6.89/3.91 (SD = 1.38/2.15)	6.57/3.27 (SD = 1.29/1.93)	2.17/4.25 (SD = 0.55/2.32)
Non-exposed	6.32/3.09 (SD = 1.18/2.00)	6.65/4.03 (SD = 1.27/2.10)	6.49/3.78 (SD = 1.21/2.04)	6.22/3.15 (SD = 1.11/1.86)	2.22/4.29 (SD = 0.58/2.20)
Differences between groups (*p*-value)	*p* < 0.001	*p* < 0.001	*p* < 0.001	*p* < 0.001	*p* < 0.001

## Data Availability

Data are available upon reasonable request from the corresponding author.

## Abbreviations

The following abbreviations are used in this manuscript:

AI	Artificial intelligence
ANCOVA	Analysis of Covariance
CCPA	California Consumer Privacy Act
CDC	Centers of Disease Control and Prevention
DPS	Digital peer support
GDPR	General Data Protection Regulation
HIPAA	Health Insurance Portability and Accountability Act
IRB	Institutional Review Board
LLM	Large Language Model
NLP	Natural Language Processing
PHI	Personal Health Information
PII	Personally identifying information
RoBERTa	Robustly Optimized BERT Pretraining Approach
SD	Standard deviation
SI	Suicidal ideation

## Appendix A

Examples provided to interpret crisis situations by Supportiv’s proprietary AI model.

A. **Active SI**
  - I feel worthless. I’m genuinely fed up with everything all the time. I can’t see my life ever getting better.
  - Honestly, I’m ready to call it a life and end it all.
  - Thoughts are overtaking me.
  - I don’t want to be here anymore. I’ve had enough.
B. **Passive SI**
  - Life makes me feel lost.
  - I don’t know. I don’t want to die because I don’t really have a bad life compared to others, but I just want to be gone. I don’t really know how to explain it. I just don’t want to feel anything or talk to anyone. I just don’t want anything really.
  - I hate my life.
  - I have a desire to die but I wouldn’t ever do anything to myself.

## Appendix B

Examples provided to interpret emotion intensities in a few-shot manner.

**Despair**

**Score 1 (low levels of despair):**

“Some stress here and there, but I can manage.”

**Score 5 (medium levels of despair):**

“I feel empty inside. I don’t care about anything anymore.”

**Score 10 (highest levels of despair):**

“I just don’t see the point anymore. It would be easier if I wasn’t here.”

**Loneliness**

**Score 1 (low levels of loneliness):**

“My best friend is the person I usually go to when I’m ranting and she rants back. We go to each other all the time for advice or for a pick me up.”

**Score 5 (medium levels of loneliness):**

“I was alone a lot at in-person things like school or clubs, but I had online friends. I can deal with my own issues alone most of the time but sometimes I do crave interaction or social support.”

**Score 10 (highest levels of loneliness):**

“My mom abandoned me when I was around five I lived with my grandmother she never let us leave the house other than going to school so I didn’t have much of a social life and I didn’t really know how to talk to other kids my age so I got bullied a lot.”

**Helplessness**

**Score 1 (low levels of helplessness):**

“I feel capable of handling challenges and making changes in my life.”

**Score 5 (medium levels of helplessness):**

“I’m trying, but I keep hitting a wall. It’s exhausting.”

**Score 10 (highest levels of helplessness):**

“Nothing will ever get better. I’ve given up. There’s no way out.”

**Depression**

**Score 1 (low levels of depression):**

“I have ordinary ups and downs, but my mood is generally stable. I enjoy most daily activities and seldom feel discouraged for more than a moment.”

**Score 5 (medium levels of depression):**

“Sadness or emptiness is present most of the day, most days. I still function, yet I must force myself. Interests start to lose their sparkle; concentration and sleep are spotty.”

**Score 10 (highest levels of depression):**

“Life feels utterly devoid of meaning. I may be unable to eat, sleep, or move without assistance. Active plans or intent to harm myself are present, and I feel incapable of keeping myself safe. Immediate professional intervention is critical.”

**Optimism**

**Score 1 (low levels of optimism):**

“Some people are just doomed no matter what they do.”

**Score 5 (medium levels of optimism):**

“Honestly, some days I wonder why I bother, but that’s when I am frustrated. I try to imagine the restart.”

**Score 10 (highest levels of optimism):**

“It restores and reminds me that there is goodness in people. The warmth and the smile show me humanity is worth every last bit of effort.”

## References

[1] World Health Organization (2025). Suicide. https://www.who.int/news-room/fact-sheets/detail/suicide.

[2] Ilic M., Ilic I. (2022). Worldwide suicide mortality trends (2000–2019): A joinpoint regression analysis. World J. Psychiatry.

[3] Center for Disease Control (2023). Suicide Rate Disparities. https://www.cdc.gov/suicide/facts/data.html#:~:text=Many%20adults%20think%20about%20suicide,1.5%20million%20attempted%20suicide.

[4] Satiani A., Niedermier J., Satiani B., Svendsen D.P. (2018). Projected Workforce of Psychiatrists in the United States: A Population Analysis. Psychiatr. Serv..

[5] American Psychological Association Psychologists Struggle to Meet Demand amid Mental Health Crisis: 2022 COVID-19 Practitioner Impact Survey. https://www.apa.org/pubs/reports/practitioner/2022-covid-psychologist-workload.

[6] Kaiser Family Foundation Mental Health Care Health Professional Shortage Areas (HPSAs). https://www.kff.org/other/state-indicator/mental-health-care-health-professional-shortage-areas-hpsas/?currentTimeframe=0&sortModel=%7B%22colId%22:%22Location%22,%22sort%22:%22asc%22%7D.

[7] Ku B.S., Li J., Lally C., Compton M.T., Druss B.G. (2021). Associations between mental health shortage areas and county-level suicide rates among adults aged 25 and older in the USA, 2010 to 2018. Gen Hosp Psychiatry.

[8] Han B., Crosby A.E., Ortega L.A.G., Parks S.E., Compton W.M., Gfroerer J. (2016). Suicidal ideation, suicide attempt, and occupations among employed adults aged 18–64years in the United States. Compr. Psychiatry.

[9] Harmer B., Lee S., Rizvi A., Saadabadi A. (2024). Suicidal Ideation. StatPearls.

[10] Wastler H.M., Khazem L.R., Ammendola E., Baker J.C., Bauder C.R., Tabares J., Bryan A.O., Szeto E., Bryan C.J. (2023). An empirical investigation of the distinction between passive and active ideation: Understanding the latent structure of suicidal thought content. Suicide Life Threat. Behav..

[11] Olgiati P., Luca M., Luca A., Ferri R., Serretti A. (2025). Passive suicide ideation in major depressive disorder: Prognostic role and effect of antidepressant treatment. J. Psychiatr. Res..

[12] Koh Y.S., Shahwan S., Jeyagurunathan A., Abdin E., Vaingankar J.A., Chow W.L., Chong S.A., Subramaniam M. (2023). Prevalence and correlates of suicide planning and attempt among individuals with suicidal ideation: Results from a nationwide cross-sectional survey. J. Affect. Disord..

[13] Howarth E.J., O’Connor D.B., Panagioti M., Hodkinson A., Wilding S., Johnson J. (2020). Are stressful life events prospectively associated with increased suicidal ideation and behaviour? A systematic review and meta-analysis. J. Affect. Disord..

[14] Chesney E., Goodwin G.M., Fazel S. (2014). Risks of all-cause and suicide mortality in mental disorders: A meta-review. World Psychiatry.

[15] Lester D., Walker R.L. (2007). Hopelessness, Helplessness, and Haplessness as Predictors of Suicidal Ideation. OMEGA—J. Death Dying.

[16] Copeland W.E., Gaydosh L., Hill S.N., Godwin J., Harris K.M., Costello E.J., Shanahan L. (2020). Associations of Despair With Suicidality and Substance Misuse Among Young Adults. JAMA Netw. Open.

[17] Shoib S., Amanda T.W., Saeed F., Ransing R., Bhandari S.S., Armiya’u A.Y.U., Gürcan A., Chandradasa M. (2023). Association Between Loneliness and Suicidal Behaviour: A Scoping Review. Turk. J. Psychiatry.

[18] Marchetti I. (2019). Hopelessness: A Network Analysis. Cogn. Ther. Res..

[19] Greer J.G., Wethered C.E. (1984). Learned Helplessness: A Piece of the Burnout Puzzle. Except. Child..

[20] Ernst M., Klein E.M., Beutel M.E., Brähler E. (2021). Gender-specific associations of loneliness and suicidal ideation in a representative population sample: Young, lonely men are particularly at risk. J. Affect. Disord..

[21] Maier S.F., Seligman M.E. (1976). Learned helplessness: Theory and evidence. J. Exp. Psychol. Gen..

[22] Gençöz F., Vatan S., Walker R.L., Lester D. (2007). Hopelessness and Suicidality in Turkish and American Respondents. OMEGA—J. Death Dying.

[23] Brunstein J.C. (1993). Personal goals and subjective well-being: A longitudinal study. J. Pers. Soc. Psychol..

[24] Ponsoni A., Branco L.D., Cotrena C., Shansis F.M., Grassi-Oliveira R., Fonseca R.P. (2018). Self-reported inhibition predicts history of suicide attempts in bipolar disorder and major depression. Compr. Psychiatry.

[25] Hawton K., Van Heeringen K. (2009). Suicide. Lancet.

[26] Coryell W., Young E.A. (2005). Clinical Predictors of Suicide in Primary Major Depressive Disorder. J. Clin. Psychiatry.

[27] Joiner T. (2007). Why People Die by Suicide. https://www.hup.harvard.edu/books/9780674025493.

[28] Chan J.K.N., Solmi M., Lo H.K.Y., Chan M.W.Y., Choo L.L.T., Lai E.T.H., Wong C.S.M., Correll C.U., Chang W.C. (2025). All-cause and cause-specific mortality in people with depression: A large-scale systematic review and meta-analysis of relative risk and aggravating or attenuating factors, including antidepressant treatment. World Psychiatry.

[29] Pointon-Haas J., Waqar L., Upsher R., Foster J., Byrom N., Oates J. (2024). A systematic review of peer support interventions for student mental health and well-being in higher education. BJPsych Open.

[30] Conversano C., Rotondo A., Lensi E., Della Vista O., Arpone F., Reda M.A. (2010). Optimism and Its Impact on Mental and Physical Well-Being. Clin. Pract. Epidemiol. Ment. Health.

[31] Carver C.S., Scheier M.F. (1998). On the Self-Regulation of Behavior.

[32] Hirsch J.K., Conner K.R., Duberstein P.R. (2007). Optimism and Suicide Ideation Among Young Adult College Students. Arch. Suicide Res..

[33] Bowersox N.W., Jagusch J., Garlick J., Chen J.I., Pfeiffer P.N. (2021). Peer-based interventions targeting suicide prevention: A scoping review. Am. J. Community Psychol..

[34] Gibson M., Moreau N., Balzamo E., Crompton D. (2023). Peer Intervention following Suicide-Related Emergency Department Presentation: Evaluation of the PAUSE Pilot Program. Int. J. Environ. Res. Public. Health.

[35] Darvishi N., Poorolajal J., Azmi-Naei B., Farhadi M. (2024). The Role of Social Support in Preventing Suicidal Ideations and Behaviors: A Systematic Review and Meta-Analysis. J. Res. Health Sci..

[36] Van Orden K.A., Witte T.K., Cukrowicz K.C., Braithwaite S.R., Selby E.A., Joiner T.E. (2010). The interpersonal theory of suicide. Psychol. Rev..

[37] Pfeiffer P.N., Heisler M., Piette J.D., Rogers M.A.M., Valenstein M. (2011). Efficacy of peer support interventions for depression: A meta-analysis. Gen. Hosp. Psychiatry.

[38] Smit D., Miguel C., Vrijsen J.N., Groeneweg B., Spijker J., Cuijpers P. (2023). The effectiveness of peer support for individuals with mental illness: Systematic review and meta-analysis. Psychol. Med..

[39] Larsen M.E., Nicholas J., Christensen H. (2016). A Systematic Assessment of Smartphone Tools for Suicide Prevention. PLoS ONE.

[40] Gould M.S., Pisani A., Gallo C., Ertefaie A., Harrington D., Kelberman C., Green S. (2022). Crisis text-line interventions: Evaluation of texters’ perceptions of effectiveness. Suicide Life Threat. Behav..

[41] Sherekar P., Mehta M. (2025). Harnessing technology for hope: A systematic review of digital suicide prevention tools. Discov. Ment. Health.

[42] Lattie E.G., Adkins E.C., Winquist N., Stiles-Shields C., Wafford Q.E., Graham A.K. (2019). Digital Mental Health Interventions for Depression, Anxiety, and Enhancement of Psychological Well-Being Among College Students: Systematic Review. J. Med. Internet Res..

[43] Kalin N.H. (2021). Anxiety, Depression, and Suicide in Youth. Am. J. Psychiatry.

[44] Torok M., Han J., Baker S., Werner-Seidler A., Wong I., E Larsen M., Christensen H. (2020). Suicide prevention using self-guided digital interventions: A systematic review and meta-analysis of randomised controlled trials. Lancet Digit. Health.

[45] Yeo G., Loo G., Oon M., Pang R., Ho D. (2023). A digital peer support platform to translate online peer support for emerging adult mental well-being: Randomized controlled trial. JMIR Ment. Health.

[46] Yeo G., Fortuna K.L., Lansford J.E., Rudolph K.D. (2025). The effects of digital peer support interventions on physical and mental health: A review and meta-analysis. Epidemiology Psychiatr. Sci..

[47] Fortuna K.L., A Naslund J., LaCroix J.M., Bianco C.L., Brooks J.M., Zisman-Ilani Y., Muralidharan A., Deegan P. (2020). Digital Peer Support Mental Health Interventions for People With a Lived Experience of a Serious Mental Illness: Systematic Review. JMIR Ment. Health.

[48] Wadden D., August T., Li Q., Althoff T. (2021). The Effect of Moderation on Online Mental Health Conversations. Proc. Int. AAAI Conf. Web Soc. Media.

[49] An Evidence-Based Model for Digital Peer Support. https://togetherall.com/en-ca/wp-content/uploads/sites/3/2023/07/Togetherall_GLOBAL_An_evidence-based_model_for_digital_peer_support_2023.pdf?.

[50] Shimgekar S.R., Rodriguez V.J., Bloom P.A., Yoo D.W., Saha K. (2025). Interpersonal Theory of Suicide as a Lens to Examine Suicidal Ideation in Online Spaces. arXiv.

[51] Vossen H.G.M., Valkenburg P.M. (2016). Do social media foster or curtail adolescents’ empathy? A longitudinal study. Comput. Hum. Behav..

[52] Brinberg M., Jones S.M., Birnbaum M.L., Bodie G.D., Ram N., Solomon D.H. (2024). How are Conversations via an On-Demand Peer-To-Peer Emotional Well-Being App Associated with Emotional Improvement?. Health Commun..

[53] Clement S., Schauman O., Graham T., Maggioni F., Evans-Lacko S., Bezborodovs N., Morgan C., Rüsch N., Brown J.S.L., Thornicroft G. (2015). What is the impact of mental health-related stigma on help-seeking? A systematic review of quantitative and qualitative studies. Psychol. Med..

[54] Patel V., Flisher A.J., Hetrick S., McGorry P. (2007). Mental health of young people: A global public-health challenge. Lancet.

[55] Obschonka M., Schmitt-Rodermund E., Silbereisen R.K., Gosling S.D., Potter J. (2013). The regional distribution and correlates of an entrepreneurship-prone personality profile in the United States, Germany, and the United Kingdom: A socioecological perspective. J. Pers. Soc. Psychol..

[56] Bailey E., Robinson J., Alvarez-Jimenez M., Nedeljkovic M., Valentine L., Bendall S., D’Alfonso S., Gilbertson T., McKechnie B., Rice S. (2021). Moderated Online Social Therapy for Young People With Active Suicidal Ideation: Qualitative Study. J. Med. Internet Res..

[57] Li T., Yang S., Wu J., Wei J., Hu L., Li M., Wong D.F., Oltmanns J.R., Wang D. (2025). Can Large Language Models Identify Implicit Suicidal Ideation? An Empirical Evaluation. arXiv.

[58] Coppersmith G., Leary R., Crutchley P., Fine A. (2018). Natural Language Processing of Social Media as Screening for Suicide Risk. Biomed. Inform. Insights.

[59] Lejeune A., Le Glaz A., Perron P.A., Sebti J., Baca-Garcia E., Walter M., Lemey C., Berrouiguet S. (2022). Artificial intelligence and suicide prevention: A systematic review. Eur. Psychiatry.

[60] Diniz E.J.S., Fontenele J.E., de Oliveira A.C., Bastos V.H., Teixeira S., Rabêlo R.L., Calçada D.B., dos Santos R.M., de Oliveira A.K., Teles A.S. (2022). Boamente: A Natural Language Processing-Based Digital Phenotyping Tool for Smart Monitoring of Suicidal Ideation. Healthcare.

[61] Reece A.G., Reagan A.J., Lix K.L.M., Dodds P.S., Danforth C.M., Langer E.J. (2017). Forecasting the onset and course of mental illness with Twitter data. Sci. Rep..

[62] Liu J., Chen J., Perrone-Bizzozero N.I., Turner J.A., Calhoun V.D. (2018). Regional enrichment analyses on genetic profiles for schizophrenia and bipolar disorder. Schizophr. Res..

[63] Wilimitis D., Turer R.W., Ripperger M., McCoy A.B., Sperry S.H., Fielstein E.M., Kurz T., Walsh C.G. (2022). Integration of Face-to-Face Screening With Real-time Machine Learning to Predict Risk of Suicide Among Adults. JAMA Netw. Open.

[64] Haroz E.E., Kitchen C., Nestadt P.S., Wilcox H.C., DeVylder J.E., Kharrazi H. (2021). Comparing the predictive value of screening to the use of electronic health record data for detecting future suicidal thoughts and behavior in an urban pediatric emergency department: A preliminary analysis. Suicide Life Threat. Behav..

[65] Elyoseph Z., Levkovich I. (2023). Beyond human expertise: The promise and limitations of ChatGPT in suicide risk assessment. Front. Psychiatry.

[66] Gutierrez G., Stephenson C., Eadie J., Asadpour K., Alavi N. (2024). Examining the role of AI technology in online mental healthcare: Opportunities, challenges, and implications, a mixed-methods review. Front. Psychiatry.

[67] A Teen Was Suicidal. ChatGPT Was the Friend He Confided. The New York Times. https://www.nytimes.com/2025/08/26/technology/chatgpt-openai-suicide.html.

[68] An AI Companion Suggested He Kill His Parents. Now His Mom is Suing. Wash Post. https://www.washingtonpost.com/technology/2024/12/10/character-ai-lawsuit-teen-kill-parents-texas/.

[69] Can A.I. Be Blamed for a Teen’s Suicide? The New York Times. https://www.nytimes.com/2024/10/23/technology/characterai-lawsuit-teen-suicide.html.

[70] Man Ends His Life After an AI Chatbot “Encouraged” Him to Sacrifice Himself to Stop Climate Change. Euro News. https://www.euronews.com/next/2023/03/31/man-ends-his-life-after-an-ai-chatbot-encouraged-him-to-sacrifice-himself-to-stop-climate-.

[71] ‘You’re Not Rushing. You’re Just Ready:’ Parents Say ChatGPT Encouraged Son to Kill Himself. CNN. https://edition.cnn.com/2025/11/06/us/openai-chatgpt-suicide-lawsuit-invs-vis.

[72] (2025). Instagram’s Chatbot Helped Teen Accounts Plan Suicide—And Parents Can’t Disable it. The Washington Post.

[73] Yeung J.A., Dalmasso J., Foschini L., Dobson R.J., Kraljevic Z. (2025). The Psychogenic Machine: Simulating AI Psychosis, Delusion Reinforcement and Harm Enablement in Large Language Models. arXiv.

[74] Holmes G., Tang B., Gupta S., Venkatesh S., Christensen H., Whitton A. (2025). Applications of Large Language Models in the Field of Suicide Prevention: Scoping Review. J. Med. Internet Res..

[75] Naddaf M. (2025). AI chatbots are sycophants—Researchers say it’s harming science. Nature.

[76] Cheng Q., Xu B., Ng M.S.N., Duan Y., So W.K.W. (2022). Effectiveness of psychoeducational interventions among caregivers of patients with cancer: A systematic review and meta-analysis. Int. J. Nurs. Stud..

[77] Moore J., Grabb D., Agnew W., Klyman K., Chancellor S., Ong D.C., Haber N. (2025). Expressing stigma and inappropriate responses prevents LLMs from safely replacing mental health providers. arXiv.

[78] OpenAI (2025). Expanding on What We Missed with Sycophancy. https://openai.com/index/expanding-on-sycophancy/.

[79] OpenAI Strengthening ChatGPT’s Responses in Sensitive Conversations. https://openai.com/index/strengthening-chatgpt-responses-in-sensitive-conversations/.

[80] McBain R.K., Cantor J.H., Zhang L.A., Baker O., Zhang F., Burnett A., Kofner A., Breslau J., Stein B.D., Mehrotra A. (2025). Evaluation of Alignment Between Large Language Models and Expert Clinicians in Suicide Risk Assessment. Psychiatr. Serv..

[81] Torous J., Firth J., Goldberg S.B. (2024). Digital Mental Health’s Unstable Dichotomy—Wellness and Health. JAMA Psychiatry.

[82] Kirtley O.J., Van Mens K., Hoogendoorn M., Kapur N., De Beurs D. (2022). Translating promise into practice: A review of machine learning in suicide research and prevention. Lancet Psychiatry.

[83] Topol E.J. (2019). High-performance medicine: The convergence of human and artificial intelligence. Nat. Med..

[84] Thakkar A., Gupta A., De Sousa A. (2024). Artificial intelligence in positive mental health: A narrative review. Front. Digit. Health.

[85] Rubin M., Li J.Z., Zimmerman F., Ong D.C., Goldenberg A., Perry A. (2025). Comparing the value of perceived human versus AI-generated empathy. Nat. Hum. Behav..

[86] Montemayor C., Halpern J., Fairweather A. (2022). In principle obstacles for empathic AI: Why we can’t replace human empathy in healthcare. AI Soc..

[87] Windler C., Clair M., Long C., Boyle L., Radovic A. (2019). Role of Moderators on Engagement of Adolescents With Depression or Anxiety in a Social Media Intervention: Content Analysis of Web-Based Interactions. JMIR Ment. Health.

[88] Perry A., Pyle D., Lamont-Mills A., Du Plessis C., Du Preez J. (2021). Suicidal behaviours and moderator support in online health communities: A scoping review. BMJ Open.

[89] Dana Z., Nagra H., Kilby K. (2024). Role of Synchronous, Moderated, and Anonymous Peer Support Chats on Reducing Momentary Loneliness in Older Adults: Retrospective Observational Study. JMIR Form. Res..

[90] Liu Y., Ott M., Goyal N., Du J., Joshi M., Chen D., Levy O., Lewis M., Zettlemoyer L., Stoyanov V. (2019). RoBERTa: A Robustly Optimized BERT Pretraining Approach. arXiv.

[91] Posner K., Brent D., Lucas C., Gould M., Stanley B., Brown G., Fisher P., Zelazny J., Burke A., Oquendo M.J.N.Y. (2008). Columbia-Suicide Severity Rating Scale (C-SSRS).

[92] Brown T., Mann B., Ryder N., Subbiah M., Kaplan J.D., Dhariwal P., Neelakantan A., Shyam P., Sastry G., Askell A. (2020). Language Models are Few-Shot Learners. arXiv.

[93] Shing H.C., Nair S., Zirikly A., Friedenberg M., Daumé H., Resnik P. (2018). Expert, Crowdsourced, and Machine Assessment of Suicide Risk via Online Postings. Proceedings of the Fifth Workshop on Computational Linguistics and Clinical Psychology: From Keyboard to Clinic.

[94] To S., Messias E., Burch L., Chibnall J. (2024). Seasonal variation in suicide: Age group and summer effects in the United States (2015–2020). BMC Psychiatry.

[95] National Institute of Mental Health (2023). Seasonal Affective Disorder. https://www.nimh.nih.gov/health/publications/seasonal-affective-disorder.

[96] Husky M.M., Olfson M., He J.P., Nock M.K., Swanson S.A., Merikangas K.R. (2012). Twelve-Month Suicidal Symptoms and Use of Services Among Adolescents: Results From the National Comorbidity Survey. Psychiatr. Serv..

[97] Nagra H., Mines R.A., Dana Z. (2025). Exploring the Impact of Digital Peer Support Services on Meeting Unmet Needs Within an Employee Assistance Program: Retrospective Cohort Study. JMIR Hum. Factors.

[98] Mahmud Shuvo S., Novely N., Faruk M.d.F., Srizon A.Y., Hasan S.M.M. (2024). Early Detection of Suicidal Ideation Using Bidirectional GRU and Language Models. Proceedings of the 3rd International Conference on Computing Advancements.

[99] Yang Z., Leonard R., Tran H., Driscoll R., Davis C. (2025). Detection of Suicidal Risk on Social Media: A Hybrid Model. arXiv.

[100] Machová K., Szabóova M., Paralič J., Mičko J. (2023). Detection of emotion by text analysis using machine learning. Front. Psychol..

[101] Abbas Q., Jeong W., Lee S.W. (2025). Explainable AI in Clinical Decision Support Systems: A Meta-Analysis of Methods, Applications, and Usability Challenges. Healthcare.

[102] Huynh A.L., Roy T.J., Jackson K.N., Lee A.G., Liaw W., Hossain M.M. (2026). Applications of artificial intelligence-based conversational agents in healthcare: A systematic umbrella review. Int. J. Med. Inf..

